# Keeping silent about emergency contraceptives in Addis Ababa: a qualitative study among young people, service providers, and key stakeholders

**DOI:** 10.1186/s12905-014-0134-5

**Published:** 2014-11-05

**Authors:** Rosalijn Both, Fantawork Samuel

**Affiliations:** Amsterdam Institute for Social Science Research (AISSR), University of Amsterdam, Amsterdam, The Netherlands; Department of Sociology, University of Addis Ababa, Addis Ababa, Ethiopia; School of Pharmacy, Addis Ababa University, Addis Ababa, Ethiopia

**Keywords:** Emergency contraceptives, Young people, Service providers, Ethiopia, Information seeking

## Abstract

**Background:**

The growing popularity of emergency contraceptives (ECs) among urban youth in Sub-Saharan Africa is accompanied by debates on morality and health. This study was situated in Addis Ababa, Ethiopia and aimed to explore how these debates affect the way in which the product is promoted at a national level, how it is dispensed by service providers, and how young people access, purchase, and get informed about ECs.

**Methods:**

Data were collected using qualitative methods: observations in pharmacies, administering semi-structured questionnaires to young people in pharmacies (N = 36), informal interviews with young people (N = 65), and in-depth interviews with service providers (N = 8) and key stakeholders (N = 3).

**Results:**

Key stakeholders, uncomfortable with high sales of ECs, and service providers, worried about women’s health, promiscuity and the neglect of condoms, stay silent about ECs. Most young people had used ECs more than once. In a context where premarital sex is morally sanctioned ECs provide young people with a way of keeping their sexual lives secret and they fit well with their sex lives that often entail infrequent sexual encounters. Young people preferred (but they are also left with no other option than) to seek information from discreet sources, including friends and partners, leaflets and the mass media. In addition, service providers misunderstood young people’s purchasing behaviour, characterized by buying ECs quickly and feeling too embarrassed to ask questions, as a rejection of counselling. The resultant lack of information about ECs sometimes led to confusion about how to take the pills.

**Conclusions:**

The attitudes and beliefs of key stakeholders and service providers result in a lack of clear information on ECs available to young people. This could be addressed by improving the information leaflet, providing clear instructions of use on blister packages, strategically distributing posters, and service providers adopting a more proactive attitude.

## Background

Recent studies from Sub-Saharan Africa show that some young people in urban areas use emergency contraceptive pills (ECs) repeatedly, sometimes as their regular contraceptive method. Repeat users claim that ECs fit into their everyday lives because they are convenient for young people who have infrequent sex, the number of pills to take is small, and they do not experience disturbing side effects [1-4]. In addition, ECs seem to be accommodated well in several West African settings where young people already use post-coital methods, and ECs can form part of a strategy for both men and women to achieve their desired reproductive goals [4].

According to recent guidelines of the World Health Organization, emergency contraceptives can be used up to 120 hours *after* intercourse; they work by preventing or delaying ovulation, and thus preventing the fertilization of an egg [5,6]. The side effects of ECs are reported to be generally mild; the most common include nausea (experienced by about 50 percent) and vomiting (experienced by about 20 percent) [5]. While ECs can be taken safely as often as needed, they are not recommended for regular use because they are less effective than other contraceptive methods and frequent use can result in menstrual irregularities [5]. They are marketed for use in emergencies *only*: when other contraceptives have failed (for example, after breakage or slippage of condoms), after incorrect use of contraceptives (for example, after missing one or more regular contraceptive pills), or after being forced or coerced into unprotected intercourse [7]. Repeat or regular use is thus considered by reproductive health experts as an ‘unintended’ way to use ECs.

When ECs are introduced into a new context, this is often accompanied by debates on morality and health. ECs are seen as having the potential to reduce the number of unwanted pregnancies and contribute to women’s empowerment, but are also often confused with abortion and associated with sexually irresponsible (promiscuous) women [6,8,9]. ECs have sparked such debates in most African countries where they have been introduced since the late 1990s or early 2000s. In Kenya, health providers and policy makers have expressed concerns about repeat EC use, claiming that the ‘e-pill’ is now the buzzword among sexually active women in Nairobi [10]. Studies among health providers in South Africa and Nigeria show widespread beliefs that repeated use poses health risks and that ECs work as an abortifacient [11,12].

The implications of these concerns about morality and health at policy and health facility level for young people’s experiences with ECs have not yet been studied. This article aims to address this gap by situating young people’s experiences with ECs in urban Addis Ababa within the context of the attitudes and beliefs of policy makers and service providers. Cross-cutting themes that appeared from the data and that will be discussed in the following sections are how ECs are perceived as an awkward and sensitive fertility regulating technology, the provision of information about ECs to young people, and what young people themselves do to seek information. First, however, it is important to outline briefly how ECs were introduced in Ethiopia.

### Emergency contraceptives in Ethiopia

In the late 1990s a national level reproductive health needs assessment concluded that ECs “could play a critical role in limiting unwanted pregnancies” in Ethiopia [13]. This report identified young women as the main beneficiaries of ECs. Over the following years, ECs were slowly introduced through youth-friendly clinics run by the Family Guidance Association of Ethiopia. No dedicated EC tablet was available in Ethiopia at that time, so packages containing four tablets of high-dose combined oral contraceptives were provided, along with simple instructions in Amharic on correct use, as well as two male condoms to encourage dual protection [14].

As a follow-up, the Ministry of Health’s Family Health Department started a two-year project in 2004 to mainstream ECs in public sector facilities [14]. During this time, young people often had to take the pills in the examination room under the supervision of a healthcare worker, to demonstrate that they had taken them correctly and would not give or sell the pills to someone else^a^. The supply of ECs in Ethiopia was accompanied by educational materials such as posters, short radio news items, and a TV commercial. However, during the commercial, the product itself was hidden and therefore unidentifiable because, the broadcasting company argued, the product was not (yet) registered and therefore could not be used in public^b^. In 2006, the Food, Medicine and Health Care Administration and Control Authority of Ethiopia (FMHACA) approved a dedicated EC product: Postinor 2. Around this time, DKT Ethiopia – a large provider of family planning products in the country – became the main supplier and distributor of ECs. Since then, ECs – under the brand name Postpill – have been made available in the private sector in pharmacies and drug stores, but this did not go hand in hand with comprehensive awareness raising about the product.

Studies have shown that Ethiopian youth prefer to buy ECs from private sector facilities instead of from public health clinics and youth-friendly services where they have been available for over a decade and where (often) more counselling is provided [2,15]. Available over the counter for 10 ETB (roughly 0.40 €), ECs are more costly than the most common condom and oral contraceptive pill brands. A recent report shows that some young people use ECs repeatedly and that pharmacists have expressed concerns that the use of ECs will lead to ‘irresponsible behaviour’ among young people and reduced condom use [2].

## Methods

The data presented in this article are part of a larger ethnographic study on sexual health technologies (in particular the use of ECs and Viagra) among young people aged 15–29 from different backgrounds in Addis Ababa, Ethiopia, and was carried out between September 2012 and January 2014. The data used for this article was gathered through observations (25 days) and the administering of semi-structured questionnaires (N = 36) in pharmacies and drug stores, and through informal interviews (N = 65) with 30 young people from different socio-economic backgrounds, as well as in-depth interviews with service providers (N = 8) and key stakeholders (N = 3).

During the first phase of the research, both authors (RB and FS) carried out observations together in two private pharmacies and one drug store, all located in densely populated neighbourhoods of Addis Ababa, over 25 days (observing approximately six hours each time), with the aim of learning which contraceptive methods are popular among young people, their purchasing behaviour, and their interactions with service providers. Initially, we randomly chose days for our observations, but soon focused on weekends and Mondays because these are popular days for EC purchases among young people. Because of the small size of the pharmacies, we were invited to sit behind the counter. This facilitated observations of the interactions between pharmacists and young people, and created opportunities for informal talks with service providers at moments when business was slow. In the drug store, FS – a young graduate of the School of Pharmacy in Addis Ababa – was asked to wear a white coat and to assist the service providers with clients. In this context, young people often approached her when purchasing contraceptives of any kind.

After purchasing contraceptives, young clients were asked by the service providers on our behalf whether they were willing to fill out a short, anonymous questionnaire about their knowledge of the contraceptive they had bought, their history of contraceptive use, and current and past interactions with service providers. When young people were willing we introduced ourselves and we made sure to explain to them that we were not part of the staff but rather independent researchers. Sixty-five young people (in the age range of 18–29) filled in the questionnaire, of which forty-eight were complete and included for data analysis. Of the usable questionnaires, thirty-six (75%) were related to the purchase of ECs and used for this article; the remainder had bought oral contraceptive pills (15%) and condoms (10%). Approximately twenty young people were unwilling to participate because they said they were in a hurry, for example because their friends or partner were waiting for them outside or calling them on their mobiles, enquiring whether they had managed to buy the ECs, or because they were visibly stressed. Sometimes we missed people who bought ECs while we were filling out the questionnaire with somebody else. Depending on young people’s preference, we filled out the questionnaire together in a separate room or at the end of the counter. FS took the lead in these conversations; RB asked the young people to elaborate on or clarify certain issues when necessary. Responses were translated on the spot by FS from Amharic to English and written down on the questionnaire (though some expressions were written down verbatim in Amharic). At the end of the conversation, many young people used the opportunity to ask us about the use and safety of ECs.

To place the findings of the observations and semi-structured questionnaires in a broader perspective, we conducted formal interviews with eight service providers working in five different pharmacies and drug stores about their experiences with young people buying ECs. These included the owners and two service providers of the two pharmacies where we did our observations and four service providers from other facilities. To assess the policy environment surrounding ECs, RB held in-depth interviews and informal interviews with three key stakeholders – the head of a social marketing organization, a representative for a leading national family planning association, and a representative of one of the major donors of EC programmes in Ethiopia – who play an important role in the funding, distribution and marketing, and administering of ECs in Ethiopia.

In the second phase of the study, a high degree of rapport was developed with 30 young men and women through snowball sampling methods, initially using our own networks selecting young people based on the criteria of being in the age range of 15–29, unmarried, having current or past experience with relationships and a willingness to talk about it, and coming from different socio-economic backgrounds. These 30 young people were involved in a total of 65 informal interviews that took place mostly in cafes, with each conversation lasting between one and two hours. The objective of following this group of young people over a longer period of time was to gain a deeper understanding of young people’s experiences with dating, relationships, and fertility regulating methods. Data from these informal interviews also provided in-depth understanding of the reasons behind the popularity of ECs, which will be discussed in a separate article [16]. The interviews were conducted by RB (when held in English) or by RB together with FS or another research assistant (when held in Amharic).

Interviews with service providers and key stakeholders were audiotaped, except for in four instances where RB took detailed notes during the conversation and typed them up in Word on the same day. None of the informal interviews with young people were audiotaped, because it was felt that this would breach the atmosphere of trust needed to talk about such sensitive issues. Instead, extensive notes were written down in a notebook immediately after the conversation and typed up in Word on the same day. Notes from the observations were also written down and typed up on the same day. Data from the questionnaires were first entered into Excel. All data sets were imported into NVivo 10 qualitative data analysis software. After carefully reading through the data, RB developed an initial codebook that was refined through discussions with FS as well as with other research assistants. Findings from observations were contrasted with what was said during the formal interviews with service providers. Cases from the semi-structured questionnaires were compared with what was said about ECs in the informal conversations. The different data collection methods used, the high degree of rapport developed with the 30 young people who participated in the informal interviews, and RB’s long-term presence in the field allowed for an in-depth understanding of EC use.

Approval for the study was obtained from the Amsterdam Institute for Social Science Research (AISSR) Ethical Advisory Board and the Medical Ethics Review Committee of the Amsterdam Academic Medical Center. In Ethiopia, social science research requires approval from Addis Ababa University, which we obtained. During the study, verbal informed consent was obtained from all participants, and they were informed that they could decline to continue at any time and that they should only talk about topics they felt comfortable with. Verbal consent was opted for over written consent because of the sensitive nature of the study topic and because of a general distrust in Ethiopia regarding the signing of documents. For the observations in pharmacies, consent was obtained from gate keepers (owner of the pharmacy/drug store and all staff members). In most pharmacies and drug stores, there is very little privacy due to their small size and because there are often several customers at the counter simultaneously. Nevertheless, we tried not to limit this privacy further by remaining seated when staff helped clients (except in the drug store where FS assisted the staff). To protect the identity and privacy of study participants, all names used in this article are pseudonyms (Tables 1 and 2).

**Table 1:** **Overview of respondents and data collection activities**

**Data**	**N**
*Observations*	
In two pharmacies and one drug store	25 days
*In*-*depth interviews*	
Key stakeholders	3
Service providers	8
*Semi*-*structured questionnaire*	
With pharmacy clients (15–29)	36
*Informal interviews*	
With young men and women (15–29)	30 (65 conversations)

**Table 2:** **Characteristics of respondents of the semi**-**structured questionnaire**

**Characteristic**	**n**	
*Sex*		
Male	16	
Female	20	
*Age*		
15-19	6	
20-24	13	
25-29	17	
*Marital status*		
Unmarried	34	
Married	2	
*Educational level*	*Male*	*Female*
Elementary (1^st^ grade - 8^th^ grade)	0	5
High school (9^th^ grade - 12^th^ grade)	6	9
Diploma	1	4
University	6	1
Unknown	3	1
*Number of times used ECs before* ^*a*^		
First time	5	
Once before	13	
2-3 times	8	
4-5 times	2	
6-10 times	2	
More than 10 times	4	

^a^It is possible that several participants underreported the number of times they had used ECs.

## Results

Among the questionnaire participants, 16 were male and 20 were female. The majority of respondents were in their twenties. All except two were unmarried, indicating the popularity of ECs among unmarried young people. The majority of participants said that they had used ECs before: 36% had used them once before, 11% had taken ECs more than ten times. The educational background of the respondents ranged from elementary school up to university level, with men being slightly more highly educated overall. Of the 30 young people who were followed over time, 8 were men and 22 were women. Five were married. Nearly half reported having used ECs at least once before. They came from different socio-economic backgrounds and included housemaids, university students, factory workers, and NGO employees. In the following sections, we first report on the views and attitudes among key stakeholders and service providers towards ECs, followed by the beliefs and practices regarding this contraceptive method among young people. We sometimes refer to ECs as the Postpill (its brand name in Ethiopia).

### Key stakeholders

Emergency contraceptives continue to occupy an awkward position within the family planning repertoire in Ethiopia and are, unlike other contraceptive methods that are actively promoted, still seen as something that should not be promoted. One key stakeholder explained:

If you tell young people about emergency contraceptives, they consider it as a regular method. You have to enable them to get information on preventive methods. This [EC] is a back-up. This is not something to promote, you have to promote preventive methods.

The same person also said that ECs are still not talked about in the public discourse in Ethiopia, and that the thousands of ECs dispensed in youth centres every year is “not something to be proud of”. Even though little was undertaken to raise awareness about ECs among young people, some key stakeholders regarded the popularity of ECs as proof of a failure to improve access to and information about *other* family planning methods.

According to the country director of a social marketing organization, advertising ECs is still a sensitive issue, not only for the government but also for the donors who fund their work. Nevertheless, the numbers of ECs sold in urban areas exceed all expectations, even without advertising:

We have seen steady growth in the sales of its EC product since introducing it in 2007. For 2013, we are on track to sell approximately 1.8 million packs of our Postpill product. This sales growth has happened with a minimal promotional effort, since one of our donors felt that our EC sales were having a negative impact on the adoption and use of ‘regular’ contraceptive methods. I suspect a more aggressive promotional effort would have resulted in even greater sales.

He therefore believed that word of mouth has played a key role in the popularity of ECs:

Since our promotional effort has been minimal (one poster and one information leaflet), it seems clear that word of mouth has been a significant driver of sales. There really is no other explanation for the level of sales that has been achieved.

### Service providers

Pharmacies and drug stores are widely available in Addis Ababa and have long opening hours: from 8:30 am until 9:30 pm. Different brands and forms of contraceptives – condoms, injectables, oral contraceptive pills, and ECs (Postpill) – are sold there. The Postpill comprises a small box containing a strip of two tablets, to be taken twelve hours apart, and a leaflet with instructions for use.

#### Frequency of sales and worries

On Sundays and Mondays (after young people have gone out at the weekend) most pharmacies sell at least ten Postpill packs. During the many religious fasting days, almost no Postpills are sold; yet very high sales occur when fasting is broken or after holidays, such as Valentine’s Day^c^. When asked about the reason for buying ECs, young people often responded they did not plan to have sex by saying ‘sex happened’. The findings from our informal interviews confirm that many young people have only occasional sex (and that this most often occurs during weekends and holidays), because they often live with their parents, because of the costs attached to spending a night in a pension, due to being involved in long-distance relationships, and because of an intense awareness about what others may think about them or their family.

Pharmacists and druggists expressed anxieties about high sales of the Postpill, saying things such as “Postpill sells like paracetamol” or expressing the wish that the Postpill was not available in Ethiopia. The country director of DKT said that he was surprised by these kinds of attitudes:

When we first introduced the product, pharmacists were extremely interested, with many of them stating that they had been wanting an EC product for many years. It’s a bit ironic that since launching the product, many pharmacists have expressed concern that the product is being overused by some women.

Service providers were worried about the possible side effects of ECs. One young female pharmacist mentioned that they could cause cancer or infertility because they contain a high dose of hormones. Furthermore, service providers often stated that, since the arrival of the Postpill, young people only care about preventing pregnancy and have forgotten about HIV/AIDS, thus neglecting condom use:

Nowadays, Postpill is highly, highly abused. Everybody every other day will use it. (…) So even the use of condoms is decreasing. Condoms are not only to protect against HIV/AIDS as youth think. Its primary means is to protect against pregnancy. When this Postpill came to our country, they may not need a condom, they think. (*Male pharmacist*)

When asked why they kept on selling the Postpill to young people despite their worries, several pharmacists hinted at the fact that pharmacies are business-oriented: if they refused to sell the Postpill to a young person, he or she would simply go and buy it elsewhere.

#### Giving advice

During in-depth interviews, service providers said that they counsel young people extensively on the use of the Postpill. Yet, in practice, most service providers would readily hand over the Postpill to young people, often telling them to take the pills twelve hours apart, but not providing them with in-depth information. Two providers admitted that they gave little advice because they were ‘afraid’ of young people who seemed unwilling to listen to them. One Monday afternoon, when we arrived at one of the pharmacies, we were told that during the morning six young people had come for the Postpill. When asked whether they had explained why they were buying it, the service provider stated:

No, sometimes even when you try to give them advice they insult you. They say ‘*min agebah*’ (what business is it of yours)? Only sometimes in the morning, when there are no others around, they will listen. (*Male service provider*)

Another pharmacist mentioned during an interview:

When teenagers come in for the Postpill, most of them do not like hearing advice. They think they know more than us. It is still a taboo to talk about sex in Ethiopia. All they say when they come in is ‘give me Postpill’. Then I start asking them when they have had sex. I can see from their facial expression that they do not want advice. So, most of the time they ask straightaway for the Postpill. They want to hide the secret. They may give me false information [regarding when they had sex] but I don’t and can’t force them to tell me the truth or tell me more. (*Male service provider*)

Our observations confirmed that young people’s purchasing behaviour, which is discussed in the following section, can easily give the impression that they are unwilling to accept advice from service providers. In addition, it is not easy to counsel young people in pharmacies and drug stores, where often more than one person is helped at the counter at the same time, leaving little room for privacy.

### Young people buying the postpill

Our observations suggest that most young people buying the Postpill are shy and poorly informed about ECs in general. Both young men (44%) and women (56%) would come to buy the Postpill. Sometimes the woman waited outside while her partner bought it for her; couples seldom came in together. In case the buyer was male, we asked him why he was buying ECs for his partner. The most common answers given by men were that their partner was too shy or scared to buy it herself, or that she was in class or at work and unable to come. One man told us that his girlfriend does not even know that she is taking the Postpill since he secretly gives it to her with a drink or banana.Young people often asked for *post* (short for Postpill), *seba hulet se*’*at* (72 hours), or *mekelakeya* (preventer). When buying the Postpill, young people would often place the exact amount of 10 ETB on the counter, implying that they knew the exact price and therefore that it was either not their first time to buy the Postpill, or that they had obtained information about it beforehand. At other times, young people bought it for a friend who was afraid to buy it. Although nearly all young people made sure that their encounter was quick and discreet, some men entered the pharmacy (seemingly) looking confident. Indeed, we observed more women than men whispering to the pharmacist, making sure there were no other clients in the facility before coming in, and running out of the pharmacy once the product had been obtained. During the informal interviews, it became clear that secrecy (and making sure not to be caught), shame, and being very conscious of what other people think are key to young people’s experiences of sexuality. The above described purchasing behaviour would seem to be a result of this.

Three times, we observed a woman coming in with a paper on which ‘emergency contraceptive’ was written:

A young girl wearing a veil and looking nervous walks into the pharmacy. She shows Fikirte [the young female pharmacist] a paper which has *dengetegna yewelid mekelakeya* (emergency contraceptive) written on it. Fikirte asks her if she means the Postpill. She shows the girl a box containing the Postpill and explains how it should be used and that it costs 10 ETB. The girl only has 8 ETB with her. Fikirte tells her to call her friend to ask if she really needs the Postpill. The girl leaves and comes back around twenty minutes later, still looking nervous. She brings 10 ETB and buys the Postpill. She asks again how she should take the two pills. Fikirte wraps it in paper for her. The girl puts the box in the pocket of her sweater and runs out of the pharmacy. (*Observational notes*, *October 2012*)

In other instances, uncertainty about how to ask for the Postpill resulted in young women receiving the regular contraceptive pill instead:

One afternoon, Fanta [co-author] is answering a phone call outside the drug store while wearing the white coat. A young woman standing outside looks into the pharmacy at the two male pharmacists behind the counter. She waits outside until Fanta (the only female service provider) finishes her call and starts talking to her. She tells her “I want a *mekelakeya* (preventer)”. Fanta asks if she wants a regular method or whether it is an emergency. The girl tells her she had sex last night. She went to a different pharmacy in the morning and asked for the same *mekelakeya*. There she was given “the strip that holds 30 pills” (regular contraceptive pills). Fanta tells her next time to ask for the “emergency” or “72 hours”. The girl had taken one oral contraceptive pill from the strip she received in the morning and asks Fanta whether it would create a problem if she took the Postpill now. Fanta explains the use of the Postpill to her. The young woman also asks Fanta whether she is in her safe days. She finished her period on Friday and had sex on Sunday. Fanta confirms that it is safe. The woman says she is scared anyway and wants to use the Postpill. She only brought 8 ETB, however; Fanta decides to pay the remaining two. (*Observational notes*, *March 2013*)

In this example, the young woman asked questions about the use of the Postpill and purposely chose to ask a peer (a young female pharmacist). This was one of the few instances in which a young woman asked questions about ECs. We never observed men asking questions to service providers. In the survey, comments such as “We usually get scared to ask questions” and “People should be able to come and ask without shame” were commonly voiced.

#### Word of mouth

Responding to the survey, 19 young people mentioned that they had learned about the Postpill from a friend through word of mouth or from their partner, and 16 said that the media was a main source of information about ECs^d^. There were no major differences between males and females. From the informal interviews it seemed that, in the absence of clear information about the Postpill, young people turned to friends or the Internet with worries or questions. Zeritu, a 23-year-old medical student, explained how a friend had asked her for advice:

The only negative thing I ever heard about the Postpill is the story of a friend who called me asking what she should do because her menstruation was flooding a lot, which was unusual. Since I used to have that problem myself, I started giving her advice and asked her how long she has had it. Then my friend told me that actually this is the first time and she was worried it might be because she took four Postpills in one month.

In addition, Mï’ïraf, a 30-year-old unmarried woman, said:

After using it [the Postpill] for like six times, I knew I was not supposed to use it every time, so I tried to find more information on the Internet about it, but I couldn’t. What to do?

The majority of survey respondents expressed a need to know more about the Postpill, especially about the possible side effects and the effects of repeated use. Phrases like ‘How often can I use the Postpill?’ were common.

The survey responses further confirmed that young people received little information from service providers: 16 mentioned that the only information they received was to take the pills twelve hours apart; nine said that they did not receive any information; and four reported that all service providers did was to warn them against repeated Postpill use.

#### Leaflet

The leaflet accompanying the Postpill has instructions for use in both Amharic and English, and turned out to be an important source of information for young people, although the content was not always considered accessible and young people sometimes disposed of the box and leaflet immediately after purchase for reasons of discretion (often inside the pharmacy, but it was also common to see empty boxes and leaflets lying on the streets of Addis Ababa). In the survey, twelve men and nine women indicated that they had read the information leaflet. They were mainly motivated by wanting to know about possible side effects (n = 10) or to know how to take it (n = 4). Eight respondents mentioned that they had never read the leaflet^e^. A few of those who had read the information leaflet mentioned that the language used was difficult to understand or that it contained confusing information, such as about the advantages of using the Postpill.^f^ This was confirmed during an informal interview with Hiwot, a 22-year-old female who had used the Postpill at least eight times:

For the Postpills, I went to different pharmacies and I asked them about the side effects of the Postpill. They all said, “well, it is this and that”. They all said different things. Then I read the leaflet and it also talks about the advantages, like that it prevents breast cancer. Then someone else says it actually causes breast cancer. So what to believe?

One 27-year-old male survey participant who bought the Postpill for his partner mentioned that he had read the information leaflet because ‘We can’t ask [questions] freely’. This comment might explain why relatively many young people read the leaflet, and underlines the importance of having a leaflet that uses clear language.

#### Buying the postpill before sex and inconsistent use

In some instances, young people wanted to buy the Postpill before having sex, to have it ready for when they needed it or in the belief that it can be taken in advance. During our observations, a female service provider told us about her experience with a housemaid the day before:

On Sunday, a housemaid who was not very confident came in and asked for the Postpill. She said that there are many boys in the house where she is working now and they have threatened her, telling her that something would happen. So she wanted to buy the Postpill in advance. I asked her why she doesn’t leave the house and go to work somewhere else. The girl said that she couldn’t leave and that her parents do not live here and that she has to work in the house. So I sold her the Postpill.

In other instances, young women were not sure when to take the Postpill or did not take the tablets correctly. A 28-year-old female survey respondent who had used the Postpill approximately ten times said:

The first time I bought it, they thought I knew. I took the two pills at the same time. Since the pharmacists were guys, I was afraid to ask.

We came across similar stories during informal interviews. For instance, 22-year-old Hiwot mentioned:

*Hiwot*: I also used the Postpill. I think I used it like eight times. But I don’t want to use it anymore (…) sometimes it didn’t work!

*RB*: How did you use it?

*Hiwot*: Normally I took it soon after sex had happened, like two, four or eight hours afterwards. One time I took the first pill. The time that I had to take the next one, twelve hours later, was at midnight. When I woke up at the time I was supposed to take it, I couldn’t find the other pill in my bag. So I didn’t take it. The next day I still didn’t have it, so I didn’t take it. Maybe I screwed it up there [soon after she discovered that she was pregnant and went for an abortion].

Mï’ïraf, a 30-year-old woman, explained how she had used the Postpill at least six times in her current relationship and how she took it incorrectly in the beginning. Even though her partner kept asking her to have a baby with him, she was not sure about the relationship and secretly used the Postpill to prevent pregnancy:

Yohannes really wanted to have a baby with me. So I started counting days, but I didn’t have a good system for that. So I used the Postpill on days we had sex, but when I wasn’t sure whether it was a safe day. The first two times I took it wrong. I took the second pill after twenty-four instead of twelve hours. Because I didn’t read the leaflet well. When I found out I used it wrongly I was so worried!

## Discussion

As in urban areas elsewhere in Sub-Saharan Africa, (repeat) use of ECs is becoming common among urban, unmarried young people in Addis Ababa. Study findings suggest that young people have occasional sex, especially during weekends and around holidays, and that ECs are a more attractive and discreet option for them than other contraceptive methods that are preferable according to reproductive health experts. The popularity of ECs occurs in a context in which the method is morally debated by key stakeholders and service providers, and where sex before marriage, though relatively prevalent, contravenes longstanding societal norms. This article focused on how the product is promoted at a national level, how providers view and dispense the product, and how young people access, purchase, and get informed about ECs.

The findings suggest that the debates on morality and health that often surround ECs also exist in Ethiopia. Ethiopia’s national family planning programme is characterized by steep increases in contraceptive prevalence rates, and the promotion and prioritization of family planning by the government as well as generous donor support are seen as having greatly contributed to this success [17]. Within this programme, oral contraceptive pills, condoms, and longer acting methods are actively promoted through different media. The study findings show that this is not the case for ECs, which continue to hold an awkward position within the family planning repertoire. Anxieties about the increasing sales of ECs and the perception that, unlike other methods, it should not be promoted have resulted in a general silence about the method among key stakeholders.

This silence was also found among service providers working in the pharmacies and drug stores where young people buy ECs. Service providers initially welcomed the product, but once confronted with the large number of young people buying ECs, they became more reluctant to sell them. They expressed worries related to negative effects on women’s health, promiscuity, and a fear that young people will forget about condoms in favour of using ECs. Similar concerns have been found among providers in other African and Asian countries, including negative opinions about repeat use, such as the belief that ECs are not meant to be used regularly and can be risky for women’s health [18]. Informally, many service providers in our study mentioned that they only continue to sell the Postpill to young people out of fear of losing clients and thus business. Service providers were also restricted by the layout of most pharmacies and drug stores, which provide little space for privacy, when it came to providing more in-depth advice and information on ECs.

Key stakeholders and service providers tend to equate ECs with (an increase in) unprotected or promiscuous sex or negative health consequences for women. In contrast, for young people, using ECs means that they can keep their sexual lives a secret in a context where premarital sex is morally sanctioned and where there is a deeply embedded culture of discretion regarding all forms of sexuality [19]. Firstly, ECs can be obtained discreetly: they are available over-the-counter in pharmacies and drug stores and men can be sent to buy them. Secondly, because the pills are consumed within 24 hours the strip can be disposed of within a day. Discarding the box and leaflet when still inside the pharmacy also ensures that their use remains covert. In addition, the fact that ECs can be taken after sexual intercourse has taken place, fits with young people’s sex lives, which they described as involving infrequent and often unplanned encounters.

Young men were actively involved in the use of ECs, particularly as purchasers of ECs to prevent their partners from feeling embarrassed or shy, and as providers of information about ECs to their partners. Other studies on EC use found how men also took on the role of monitor (ensuring that their partners took their pills correctly), and that of provider of emotional support [2,4]. Young men thus take on supportive roles in their girlfriends’ use of ECs. Yet the example of a young man who secretly gave the Postpill to his girlfriend raises questions about the frequency of and motivations behind instances in which partners use the product without the other knowing and requires further study.

Young people’s need for discreetness and key stakeholders’ and service providers’ (moral) silence affects young people’s access to information about ECs in two important ways. Firstly, it leads to misunderstandings between young people and service providers. Young people do not want to be seen buying ECs so they hurriedly purchase the product. Service providers therefore get the impression that they already know the product or would not accept counselling. On the contrary, however, the findings suggest that young people have many questions about ECs, especially about side effects and how to use them, but often feel too shy or ashamed to raise these issues with service providers. Secondly, given the absence of clear information about ECs, young people are left with no other choice than to seek information from discreet (often informal) channels. These include learning from peers and partners (word of mouth), reading leaflets, and to a lesser extent searching the Internet. A study in Uganda among out-of-school adolescents showed how peers were the most important, though not preferred, source of information on sexual and reproductive health issues, including contraceptives. The reliability of the information, pressure from peers to be sexually active, and fear among girls that peers may be indiscreet with personal information were frequently voiced concerns [20]. Similarly, although young people in this study relied heavily on friends and partners for knowledge about ECs, they expressed a need for more and clearer information [2].

The findings indicate that key stakeholders, sexual and reproductive health and rights organizations, and service providers need to break the silence around ECs through the use of effective and discreet information delivery channels. Revising the information leaflet into an attractive and clear reference document, possibly including illustrations to accommodate people of all literacy levels, is one way to better inform young people. Young people often consult the leaflet accompanying the Postpill, but complain that the language used is too technical and that it is sometimes confusing. Elsewhere, it has been argued that leaflets deliberately use small type fonts to ‘hide’ information about a product’s side effects [21]. As with the oral contraceptive pill, instructions for use or key messages could also be printed on the blister package itself [22]. This is likely to be effective, since we observed several young people throwing away the Postpill box and leaflet immediately after purchase, for the reasons mentioned above.

Posters with clear and short messages about ECs could be placed in and outside of pharmacies. Messages should address the issues that are most pressing for young people: the effects of repeated use and side effects. Other messages should highlight that ECs only offer protection against pregnancy but not against HIV, and suggest alternatives. Furthermore, the possibility of providing condoms along with the sale of ECs should be considered. These messages could also be made available at places where young people hang out, such as schools and universities, as well as at pensions where young people often spend the night and in bars and nightclubs. Sexual and reproductive health and rights organizations should make use of the knowledge that sales of ECs fluctuate and are highest during the weekends and on Mondays, and around national religious holidays (when fasting is broken) and international holidays (such as Valentine’s Day), by implementing targeted campaigns during such times.

Service providers should not be discouraged by young people’s purchasing behaviour, nor should they base their advice on their own misconceptions or negative opinions about ECs. Pharmacies and drug stores are not ideal places to provide young people with information and young people may purposely visit these places to avoid the extensive counselling that they receive at other facilities. We nevertheless observed how service providers often did manage to instruct young people to take the tablets twelve hours apart. They could additionally advise young people to read the information leaflet and encourage them to ask questions.

Although somewhat out of the scope of our research, other discreet communication channels such as the Internet and possibly mobile phones could be used to inform young people about ECs. Reaching young people with information on contraceptives through mobiles phones in Kenya and promoting condom sales and use through a mixture of digital platforms – including Facebook – in Turkey have proven to be effective [23,24].

### Strengths and limitations

The findings of this study are based on a small sample size and cannot be generalized to the whole of Ethiopia (especially not to rural areas: ECs may be not or only limited available in those areas and the context of dating, relationships and the use of fertility regulating methods differ) but it is most likely that similar issues are at stake in other urban areas of Ethiopia [2]. The results also coincide with other countries where studies similarly found moral attitudes among key stake holders and service providers, and showed that ECs fit into young people’s lives [2-4,18]. Due to the sensitivity of the topic, it is possible that some young people, especially those whom we approached in the pharmacies (where there was little opportunity for rapport building), underreported the number of times they had used ECs.

## Conclusions

The study findings illuminate how national level and health provider attitudes affect the type and amount of reproductive health information available to young people, as well as the different tactics employed by young people to inform themselves about fertility regulating methods. The findings should be taken into consideration in service provision in pharmacies and drug stores, and key stakeholders should reflect on how their attitudes affect how young people access and use ECs. Keeping silent about ECs is not of benefit to young people, who need clear and discreetly offered information on their use.

## Endnotes

^a^Informal conversation with a respondent from a leading national family planning association in Ethiopia, May 2013.

^b^Ibidem.

^c^Ethiopian Orthodox Christianity, the dominant religion in the country, has up to 110–150 fasting days a year. Fasting entails restrictions on the time of consumption of food, the diet allowed, and abstinence from sexual activity.

^d^Unfortunately, we did not ask them to specify the media source.

^e^Eight people did not answer this question, some because they were buying the Postpill for the first time.

^f^The leaflet has a separate paragraph entitled ‘Non-contraceptive benefits’ that talks about the beneficial effects of ECs, including a reduction in the incidence of benign breast cancer.
